# Supplementary material for: Vegetation Formation in Staphylococcus Aureus Endocarditis Inversely Correlates With RNAIII and sarA Expression in Invasive Clonal Complex 5 Isolates
Source: Front Cell Infect Microbiol. 2022 Jul 4;12:925914. doi: 10.3389/fcimb.2022.925914 (PMC9289551; doi:10.3389/fcimb.2022.925914)
Supplement: Supplementary file 1 [file DataSheet_1.docx]

Supplementary Material

# Supplementary Figures and Tables

## Supplementary Figures

# Figure S1. Current understanding of *S. aureus* global regulator control of gene expression. The Agr quorum-sensing system differentially controls expression of *S. aureus* surface proteins [e.g. microbial surface components recognizing adhesive matrix molecules “(MSCRAMMs) and protein A] and the secreted toxins and enzymes (e.g. hemolysins, superantigens, proteases, nucleases, lipases) (Jenul and Horswill, 2019). RNAIII is the effector molecule of the Agr system and its expression is regulated by AgrA. When the Agr system is inactive, *rot* (for *repressor of toxins*) is expressed. Rot induces SarS and surface proteins and reduces SaeRS and secreted toxins and enzymes. When the Agr system is active, *RNAIII* is expressed and Rot is translationally suppressed. Inhibition of Rot allows for expression of the secreted toxins and enzymes and concomitantly reduces expression of SarS and surface proteins (Mcnamara et al., 2000; Saïd-Salim et al., 2003; Geisinger et al., 2006). SarA increases expression of the *agr* system (Heinrichs et al., 1996; Rechtin et al., 1999) and genes encoding for the phenol-soluble modulins (PSMs) (Morrison et al., 2012) and represses expression of *sarS* and *rot* (Cheung et al., 2001; Hsieh et al., 2008). SarS acts opposite to SarA by inducing expression of *spa* (Protein A) and repressing expression of several toxin genes such as *hla* (α-toxin gene) (Tegmark et al., 2000). The alternative sigma factor B (SigB) directly inhibits expression of the *agr* operon (Bischoff et al., 2001) and of *rot* during growth in stationary phase (Hsieh et al., 2008), but induces expression of *sarA*, adhesin genes and the enterotoxin gene cluster (*egc*) (Bischoff et al., 2004; Entenza et al., 2005; Kusch et al., 2011). MgrA regulatory effects mirror those of the *agr* system, as RNAIII stabilizes mgrA when derived from the P2 promoter, although the specific targets can be distinct (Luong et al., 2006; Gupta et al., 2015). It has also been reported that MgrA regulates *agr* expression in some strains (Jenul and Horswill, 2019). MgrA upregulates production of secreted proteins (e.g. leukotoxins, Spl proteases, enzymes) and downregulates production of surface-associated proteins (e.g. large surface protein Ebh, SraP, SasG) (Luong et al., 2006; Crosby et al., 2016).


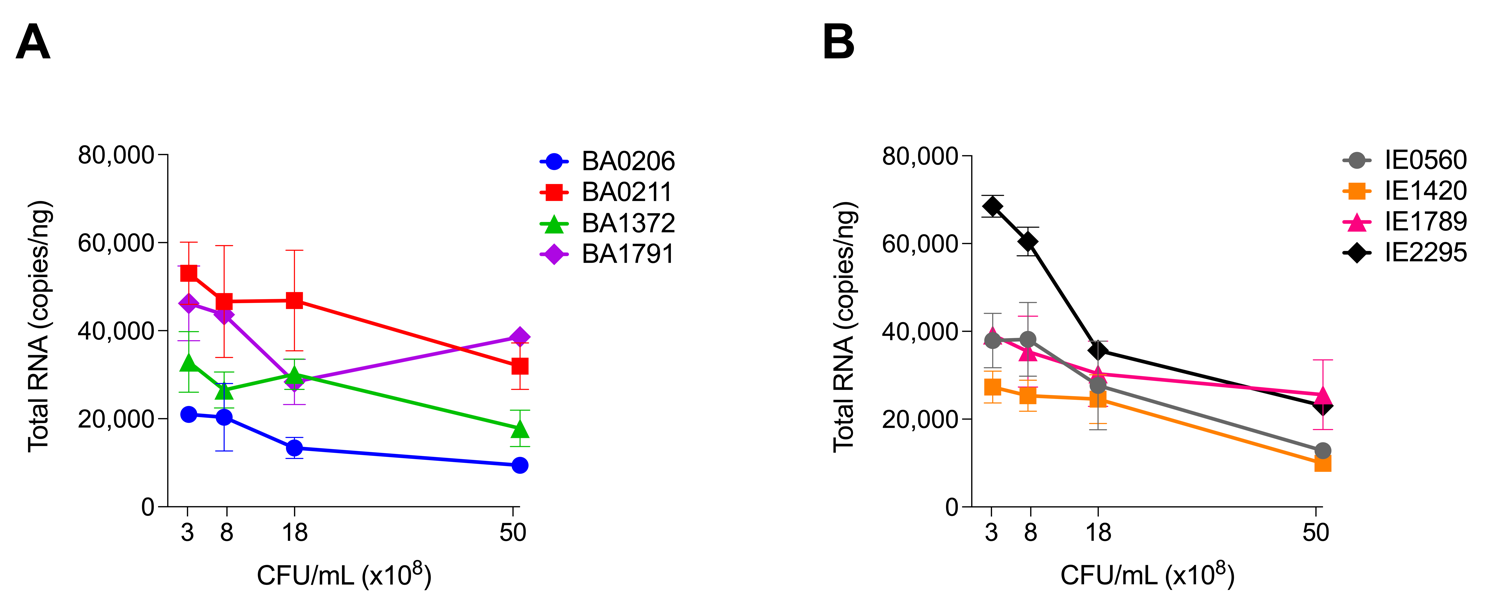


**Figure S2. CC5 clonal group differentially express *gyrB*.** (**A, B**) Quantitation of *gyrB* mRNA in *S. aureus* CC5 during growth in TH broth by RT-qPCR standard curve quantitation method. Data is represented by the mean ± SEM of three biological replicates. Error bars (standard deviation) not shown are smaller than symbol. Two-way ANOVA with Holm-Šídák's multiple comparisons test.

## Supplementary Tables

**Table S1. Kidney gross pathology grading scale**

| **Kidney Pathology** | | |
| --- | --- | --- |
| Color: red/hemorrhagic (+/-)  white/grey/necrotic (+/-)  mottled red/grey (+/-) | 0 | No lesions |
|  | 1 | Rare, up to 4-5 small (<4mm) multifocal lesions (infarcts) on surface |
|  | 2 | Numerous larger (>5mm) multifocal lesions (infarcts) on surface |
|  | 3 | Locally extensive to coalescing to diffuse lesions (infarcts) on surface |

**Table S2. RT-qPCR Primers**

| **Primer** | **Primer Sequence** |
| --- | --- |
| gyrB_RTqPCRFwd  gyrB_RTqPCRRev | CGTCCAGCTGTCGAAGTTATT  CTGATGAACCAACACCATGTAAAC |
| RNAIII_RTqPCR_For  RNAIII_RTqPCR_Rev | GCACTGAGTCCAAGGAAACT  AGCCATCCCAACTTAATAACCA |
| rot_RTqPCR_For  rot_RTqPCR_Rev | GCATTGCTGTTGCTCTACTTG  CGTCCTGTTGACGATGAAAGA |
| mgrA_RTqPCR_For  mgrA_RTqPCR_Rev | TCGGAACGTTCACGCTTAAT  TCTCCTGTAAACGTCAAGAAAGT |
| SigB_RTqPCR_For  SigB_RTqPCR_Rev | TCGCGAACGAGAAATCATACA  CCGTTCTCTGAAGTCGTGATAC |
| SarA_RTqPCR_For  SarA_RTqPCR_Rev | TAGCTTTGAAGAATTCGCTGTATTG  GCTTTAACAACTTGTGGTTGTTTG |
| SarS_RTqPCR_For  SarS_RTqPCR_Rev | CGCTCAACTGAAGATGAAAGAAA  TGAGCTAATAATTGTTCAGCATGG |
| SEG RT-qPCR For  SEG RT-qPCR Rev | ACTGTACAGGTAACAATCGACAATAG  TGCAGAACCATCAAACTCGTATAG |
| SEl-I RT-qPCR For  SEl-I RT-qPCR Rev | GGGCCACTTTATCAGGACAATAC  ACATCAATTTCTTGAGCTGTGACTA |
| SElM_N315_RTqPCR_For  SElM_N315_RTqPCR_Rev | GGTGGAGTTACATTAGCAGGTG  TGATGTTCTCCATTAACCCAAAGA |
| SEl-N RT-qPCR_ For  SEl-N RT-qPCR_ Rev | GGACTGTATTATGGAAATAAATGTGTAGGC  ACCTTCTTGTTGGATACCATCTT |
| SEO_N315_RTqPCR_For  SEO_N315_RTqPCR_Rev | TTTAGCTCATCAGCGATTTCTAAAG  CCACCATATGTACAGGCAGTAT |
| SEl-U_RTqPCRFwd  SEl-U_RTqPCRRev | GTGTTAAGTCTTGCAGCTTACTATT  CCTCATATTATCCATTAGACCAGTGA |
| SEl-X_RTqPCRFwd  SEl-X_RTqPCRRev | TCTATCGCTAGGTATCATCTATGGG  GGAATTGTTTATCTTGTACACTTGGG |

**Table S3. qPCR assay performance**

| **Target Amplicon** | **Amplicon Length (bp)** | **Melt Curve Tm (SD)** | **PCR Efficiency (%)** | **R^2^ Calibration Curve** | **Linear Dynamic Range (Copies)** | **Intraassay Variance**  **(SD of C_q_)** |
| --- | --- | --- | --- | --- | --- | --- |
| gyrB | 103 | 80.56 (0.28) | 97.73 | 0.999 | 100-1,000,000 | 0.06 |
| RNAIII | 81 | 76.69 (0.15) | 103.5 | 0.998 | 100-10,000,000 | 0.08 |
| rot | 118 | 77.99 (0.14) | 101.1 | 0.996 | 100-1,000,000 | 0.06 |
| mgrA | 113 | 78.22 (0.14) | 99.02 | 0.999 | 100-1,000,000 | 0.09 |
| sigB | 110 | 79.34 (0.15) | 99.84 | 0.999 | 100-1,000,000 | 0.12 |
| sarA | 114 | 75.22 (0.15) | 97.65 | 0.999 | 100-1,000,000 | 0.08 |
| sarS | 80 | 77.32 (0.14) | 99.33 | 0.999 | 100-1,000,000 | 0.09 |
| seg | 147 | 77.56 (0.17) | 98.92 | 0.998 | 10-1,000,000 | 0.04 |
| sei | 139 | 77.27 (0.17) | 100.5 | 0.999 | 10-1,000,000 | 0.06 |
| sem | 80 | 74.72 (0.15) | 101.3 | 0.998 | 10-1,000,000 | 0.07 |
| sen | 150 | 77.37 (0.21) | 102.6 | 0.998 | 10-1,000,000 | 0.06 |
| seo | 119 | 78.08 (0.12) | 98.41 | 0.998 | 10-1,000,000 | 0.13 |
| se*l*u | 101 | 76.83 (0.05) | 96.4 | 0.998 | 100-1,000,000 | 0.10 |
| se*l*x | 98 | 78.51 (0.14) | 101.3 | 1.000 | 100-1,000,000 | 0.09 |
